# Interpretable admission-stage machine learning for early Sepsis-3 identification in the emergency department

**DOI:** 10.3389/fmed.2026.1929426

**Published:** 2026-09-18

**Authors:** Fulden Cantaş Türkiş, Buğra Varol

**Affiliations:** Division of Biostatistics, Faculty of Medicine, Muğla Sıtkı Koçman University, Muğla, Türkiye

**Keywords:** clinical classification model, clinical decision support, emergency department, explainable artificial intelligence, interpretable machine learning, sepsis

## Abstract

**Background/objectives:**

Artificial intelligence-based clinical decision support systems have emerged as promising tools for improving early sepsis recognition in emergency departments. This study aimed to develop and evaluate interpretable machine learning models for identifying Sepsis-3-defined sepsis using routinely available admission-stage variables and to compare their performance with conventional biomarkers and the Systemic Inflammatory Response Syndrome (SIRS).

**Methods:**

This secondary analysis used a publicly available prospective cohort of 1,572 adult emergency department patients with suspected community-onset sepsis. The outcome was Sepsis-3-defined sepsis. Thirteen demographic, physiological, and laboratory admission variables were evaluated. Following a stratified 70:30 train–test split, missing data were handled using multivariate imputation by chained equations, and feature selection was performed using least absolute shrinkage and selection operator logistic regression. Penalized logistic regression (LR), Explainable Boosting Machine (EBM), Extreme Gradient Boosting (XGBoost), and Light Gradient Boosting Machine (LightGBM) models were developed using five-fold cross-validation. Performance was evaluated by discrimination, classification, calibration, decision curve analysis, and explainability.

**Results:**

Among 1,572 adult patients, 560 met the Sepsis-3 criteria. In the internal holdout test set, AUROC values were 0.753 (LR), 0.763 (XGBoost), 0.767 (LightGBM), and 0.766 (EBM). Conventional biomarkers and SIRS showed lower discrimination (AUROC 0.599–0.693), whereas a biomarker-only logistic regression model achieved an AUROC of 0.710. No significant AUROC differences were observed among the machine learning models. LightGBM achieved the numerically highest AUROC and accuracy, whereas EBM achieved the highest sensitivity (0.845). Age, procalcitonin, oxygen saturation, respiratory rate, and neutrophil-to-lymphocyte ratio were the most influential predictors.

**Conclusions:**

Interpretable machine learning models based on routinely available emergency department admission variables provided moderate discrimination, clinically relevant sensitivity, and transparent predictor effects for identifying Sepsis-3-defined sepsis. Compared with conventional biomarkers, SIRS, and a biomarker-only logistic regression model, the machine learning models generally showed higher discrimination, although the magnitude and statistical significance of these differences varied across comparators. External validation is required before clinical implementation.

## Introduction

Sepsis is a major cause of preventable morbidity and mortality worldwide and remains one of the most time-sensitive conditions encountered in emergency care (1–3). According to the Third International Consensus Definitions for Sepsis and Septic Shock (Sepsis-3), sepsis is life-threatening organ dysfunction caused by a dysregulated host response to infection and is operationalized by an acute increase of at least two points in the Sequential Organ Failure Assessment (SOFA) score (4). Because emergency department (ED) clinicians must often make early decisions before microbiological confirmation is available, timely recognition of patients at risk for sepsis remains a major clinical challenge. Contemporary guidelines emphasize rapid identification and early management; quick SOFA (qSOFA) was introduced as a bedside clinical criterion in the Sepsis-3 framework, although current guidelines caution against relying exclusively on qSOFA as a single screening tool (3, 5).

Several clinical signs and biomarkers are routinely used to support sepsis assessment, including vital signs, leukocyte indices, C-reactive protein (CRP), procalcitonin, and lactate. However, these markers may overlap between infectious and non-infectious inflammatory conditions, and their individual diagnostic performance is often limited (6, 7). In the original Skaraborg Hospital cohort of adults presenting to the ED with suspected bacterial sepsis, procalcitonin, neutrophil-to-lymphocyte ratio (NLR), CRP, and lactate were evaluated individually and in combination; for Sepsis-3-defined bacterial sepsis, procalcitonin achieved an area under the receiver operating characteristic curve (AUROC) of 0.68, whereas composite biomarker models showed higher discrimination mainly in more severe Sepsis-2 categories such as severe bacterial sepsis or septic shock (8). These findings suggest that single biomarkers alone may be insufficient for reliable early ED risk stratification and support the need for multivariable approaches that integrate clinical, physiological, and laboratory information available at admission.

Machine learning has increasingly been investigated as a strategy to improve sepsis prediction by capturing nonlinear relationships and interactions among routinely collected clinical variables (9). ED-based studies have developed machine learning risk scores and gradient boosting models for sepsis identification and compared them with conventional sepsis and early-warning scores such as systemic inflammatory response syndrome (SIRS), SOFA, qSOFA, Modified Early Warning Score (MEWS), and National Early Warning Score (NEWS) (10, 11). Other approaches have used unstructured triage information and natural language processing (NLP), reporting substantial performance gains when free-text data are added to structured vital signs and demographic variables (12, 13). Although these studies demonstrate the potential of machine learning in ED sepsis prediction, many models rely on broad electronic health record (EHR) data, unstructured text, local operational definitions, or advanced informatics infrastructure that may not be readily available across different clinical settings.

Artificial intelligence (AI) is increasingly being integrated into clinical decision support (CDS) systems to assist clinicians in diagnostic reasoning, risk stratification, and treatment prioritization. In emergency medicine, where rapid decisions must often be made before confirmatory testing becomes available, interpretable AI-based decision support tools may improve consistency while maintaining clinician oversight.

Interpretability is another important requirement for clinical translation. Black-box predictions may be difficult for clinicians to trust, particularly in high-risk and time-sensitive conditions such as sepsis. Interpretable machine learning models and *post hoc* explanation techniques can help clarify how individual predictors contribute to model output and whether model behavior is clinically plausible (14, 15). At the same time, predictive performance should not be judged by discrimination alone; calibration and clinical utility are essential for evaluating whether predicted risks are reliable and whether a model provides net clinical benefit across decision thresholds (16–19).

A recent study using the same open-access Skaraborg ED cohort developed an Explainable Boosting Machine (EBM)-based prediction model for Sepsis-3-defined sepsis and reported high discriminative performance after class balancing with Synthetic Minority Oversampling Technique for Nominal and Continuous variables (SMOTE-NC) and feature selection procedures (20). The study identified positive blood culture, oxygen saturation, procalcitonin, age, and NLR as the most influential predictors of sepsis. While these findings further support the potential of interpretable machine learning approaches in emergency care, some of the most influential variables, particularly positive blood culture, may not be available at the time of initial clinical assessment and are closely related to established infection. Therefore, there remains a need for clinically pragmatic identification models that rely exclusively on routinely available admission variables, avoid outcome-proximal information, preserve the original outcome distribution as much as possible, and provide transparent explanations of model behavior.

The present study was designed to address this gap. The contribution of the present study lies in a clinically focused reanalysis of the Skaraborg cohort using a restricted set of admission-stage variables, exclusion of outcome-proximal information, comparison of multiple machine learning algorithms within a consistent analytical framework, and evaluation of calibration, clinical utility, and explainability. Using a publicly available prospective ED cohort of adults with suspected community-onset sepsis, penalized logistic regression (LR), Explainable Boosting Machine (EBM), Extreme Gradient Boosting (XGBoost), and Light Gradient Boosting Machine (LightGBM) models were developed and compared for identifying Sepsis-3-defined sepsis. The models were intentionally restricted to predefined demographic, physiological, and laboratory variables routinely available at ED admission, thereby reflecting a clinically pragmatic AI-based CDS framework. Model development incorporated training-based imputation, Least Absolute Shrinkage and Selection Operator (LASSO) feature selection, internal holdout validation, calibration assessment, decision curve analysis (DCA), and complementary explainability using EBM and SHapley Additive exPlanations (SHAP). In addition to comparisons with conventional biomarkers and SIRS, the incremental predictive value of incorporating routinely available admission-stage clinical variables beyond a biomarker-only logistic regression model was evaluated. The aim was to determine whether a compact, clinically interpretable, and admission-stage machine learning framework could provide reliable early sepsis risk stratification while maintaining transparency and potential clinical applicability in the ED.

## Methods

This secondary analysis was designed as a clinical classification modeling study using a publicly available prospective ED cohort. The methodological workflow included outcome definition, stratified data partitioning, missing-data imputation, feature selection, supervised machine learning model development, internal holdout validation, calibration and clinical utility assessment, and complementary explainability analyses. To minimize information leakage, all preprocessing, feature selection, hyperparameter tuning, threshold determination, and model training procedures were restricted to the training data before final evaluation in the internal holdout test set. An overview of the study design and analytical workflow is presented in Figure 1.

**Figure 1:**
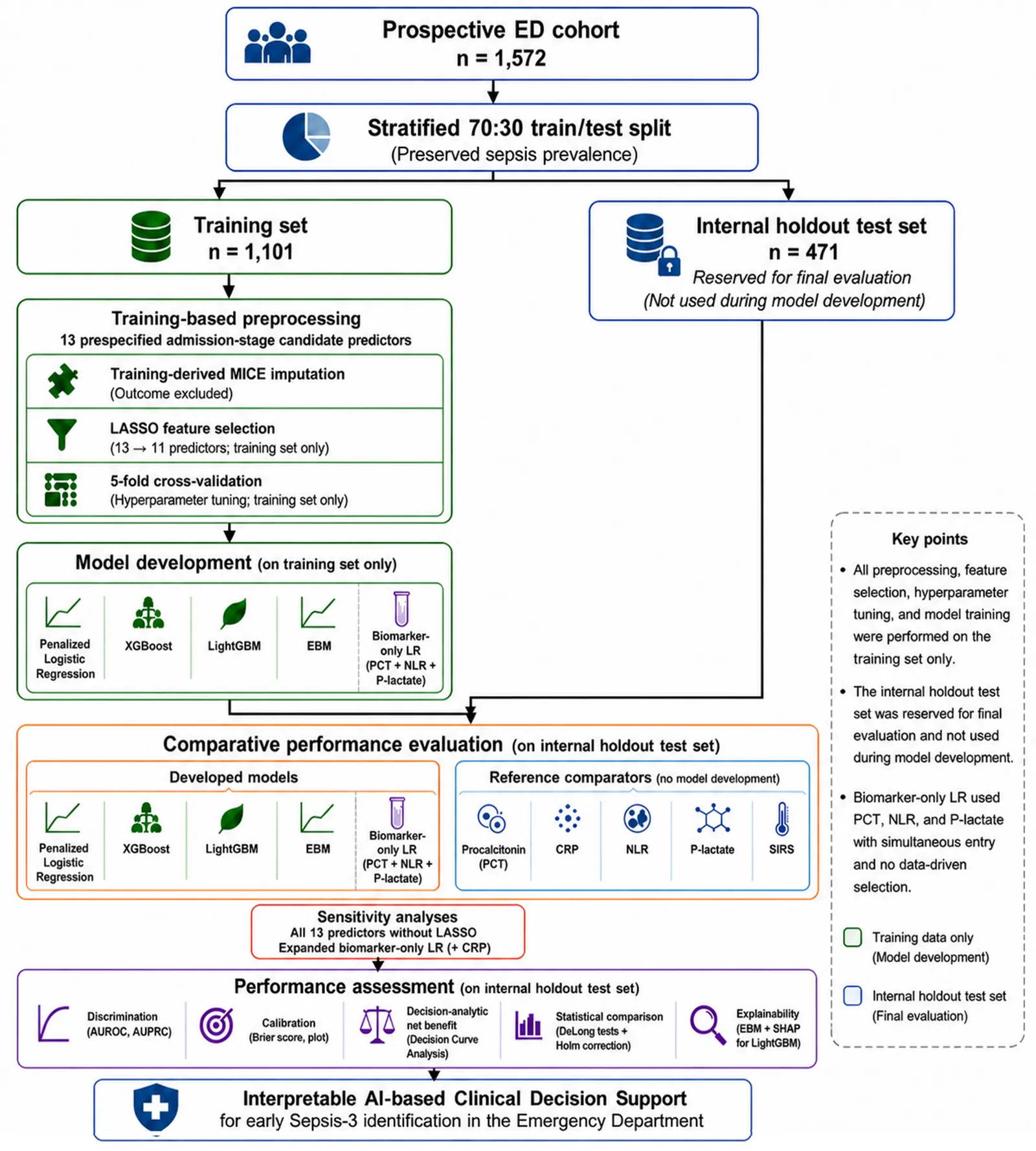
Overview of the study design, model development, and evaluation workflow.

### Study design and population

This study was conducted as a secondary analysis of a previously published prospective observational cohort comprising adult patients presenting to the ED with suspected community-onset sepsis at Skaraborg Hospital, Sweden, between September 2011 and June 2012 (8). The original cohort included 1,572 adult patients with suspected community-onset sepsis, representing 1,572 unique individuals. Demographic characteristics, clinical findings, physiological measurements, and laboratory parameters were collected at admission before initiation of antibiotic treatment.

For the present investigation, the publicly available dataset was reanalyzed to develop and validate machine learning models for identifying patients meeting the Sepsis-3 definition. Patients were classified according to the Sepsis-3 definition, in which sepsis is defined as infection-associated organ dysfunction represented by an increase of at least two points in the SOFA score (4). Within the study cohort, 560 patients fulfilled the Sepsis-3 criteria, whereas 1,012 patients were classified as non-septic. The intended target population of the present analysis was therefore adults presenting to the ED with clinically suspected community-onset sepsis, rather than an unselected ED population. The non-sepsis group comprised patients who did not meet the Sepsis-3 definition, including patients with verified bacterial infection without Sepsis-3-defined organ dysfunction as well as patients with possible but non-verified bacterial infection, viral infection, or non-infectious conditions.

The original study protocol was approved by the Regional Ethical Review Board of Gothenburg, Sweden, and written informed consent was obtained from all participants prior to enrollment (8). Because the present study involved secondary analysis of anonymized publicly available data, additional ethical approval was not required.

### Outcome definition

The primary outcome of this study was the presence of sepsis according to the Sepsis-3 definition. Consistent with the Sepsis-3 definition, sepsis was defined as infection-associated organ dysfunction characterized by an increase of at least two points in the SOFA score (4). Predictor variables were obtained at ED admission, whereas Sepsis-3 status was determined from the outcome classification provided in the original cohort annotations. Patients meeting the Sepsis-3 criteria were assigned to the sepsis group, whereas those not meeting these criteria were assigned to the non-sepsis group. Consequently, the task was formulated as a binary classification problem aimed at distinguishing patients with Sepsis-3 from those without Sepsis-3 using demographic, clinical, physiological, and laboratory variables available at ED admission. Among the 1,572 patients included in the dataset, 560 were classified into the sepsis group and 1,012 into the non-sepsis group.

### Data partitioning

The dataset was randomly divided into a training set (70%) and an internal holdout test set (30%) using stratified sampling to preserve the proportion of sepsis and non-sepsis patients across both datasets. The training set was used for all model development procedures, whereas the internal holdout test set was reserved exclusively for final model evaluation.

### Candidate predictors

Although the original dataset contained a larger number of clinical and laboratory variables, 13 candidate predictors were selected *a priori* for model development based on clinical relevance, routine availability during the initial ED assessment, and supporting evidence from the sepsis literature. Variables collected after ED admission, variables closely related to subsequent outcome determination, and variables not routinely available during the initial ED assessment were not considered as candidate predictors. The predefined candidate predictors were age, sex, systolic blood pressure (SBP), respiratory rate, oxygen saturation, heart rate, body temperature, hemoglobin, leukocyte particle concentration, CRP, procalcitonin, NLR, and plasma lactate concentration (P-lactate).

### Missing data handling

Missing data were handled using multivariate imputation by chained equations (MICE) after the stratified train–test split. Twenty imputed datasets were generated (*m* = 20), with 20 iterations per chain. Predictive mean matching was used for incomplete continuous variables, including systolic blood pressure, respiratory rate, oxygen saturation, heart rate, body temperature, hemoglobin, leukocyte particle concentration, C-reactive protein, neutrophil-to-lymphocyte ratio, and P-lactate. Age, sex, and procalcitonin contained no missing values and therefore were not imputed.

Each incomplete variable was imputed using the remaining admission-stage candidate predictors, while patient ID, Sepsis-3 outcome status, and the train–test indicator were excluded from the imputation models. The outcome variable was neither imputed nor used as a predictor. To prevent information leakage, test-set observations were excluded from estimation of the conditional imputation models and therefore did not contribute to the imputation-model parameters. Missing predictor values in the test set were subsequently imputed using the models estimated from the training data and the available predictor information for each test observation, without use of test-set outcome information.

For the downstream machine-learning analysis, a single representative completed dataset was selected from the 20 imputations. A multivariable logistic regression model was fitted to each imputed training dataset, and the regression coefficients were pooled using Rubin's rules. The root-mean-square distance between each imputation-specific coefficient vector and the pooled coefficient vector was then calculated, and the completed dataset with the smallest distance was selected for subsequent feature selection, model development, and internal holdout evaluation. The selection was based only on the training data and did not use test-set outcomes or downstream machine-learning performance. Accordingly, the reported machine-learning performance estimates were obtained from this representative completed dataset rather than pooled across all 20 imputations. As a sensitivity analysis, five of the 20 completed datasets were randomly selected, and the complete modeling pipeline, including LASSO feature selection, hyperparameter tuning, model fitting, and internal holdout test-set evaluation, was repeated independently in each selected dataset. Model stability across imputations was assessed using AUROC and Brier score. Convergence was assessed using MICE chain diagnostics. The chains showed adequate mixing without persistent trends or systematic separation, and no events were recorded in the MICE logged-event output.

### Feature selection

Feature selection was performed using the LASSO logistic regression method. LASSO was used as a prespecified feature-selection step to derive a compact predictor set and limit unnecessary model complexity before subsequent model development. LASSO applies an L1 penalty to regression coefficients, shrinking less informative predictors toward zero and thereby reducing model complexity and overfitting. The optimal penalty parameter (*λ*) was identified through five-fold cross-validation within the training dataset. Predictors with non-zero coefficients at the selected *λ* value were retained for subsequent model development (21).

### Final modeling dataset

Continuous predictors were entered into the models in their original scale without transformation or standardization. Following imputation and feature selection, the retained predictors were used for the development and validation of all machine learning models. The same predictor set was employed across all algorithms in the primary analysis to facilitate direct and consistent comparison of predictive performance.

### Machine learning model development

Four supervised machine learning algorithms were developed to identify Sepsis-3 status: LR, EBM, XGBoost, and LightGBM. Model development was performed exclusively within the training dataset. For each algorithm, hyperparameter optimization was conducted using a grid search strategy combined with five-fold cross-validation. The same cross-validation folds were used throughout the optimization process to ensure consistency and comparability across models. Hyperparameter combinations yielding the highest mean AUROC during cross-validation were selected as the optimal configurations. No information from the internal holdout test set was used during feature selection, hyperparameter optimization, threshold determination, or model training.

Following hyperparameter optimization, the optimal model for each algorithm was retrained using the entire training dataset. The internal holdout test set remained completely unseen during model development and was used solely for final performance evaluation.

All machine learning models in the primary analysis were developed using the same set of predictors and an identical preprocessing pipeline to facilitate direct comparison of predictive performance. No class weighting, oversampling, or undersampling was applied to any of the models.

### Model validation and performance assessment

Model performance was evaluated using the internal holdout test set. The primary measure of discrimination was the AUROC. In addition, classification performance was assessed using accuracy, sensitivity, specificity, *F*1-score, and the area under the precision–recall curve (AUPRC).

For each model, the optimal probability threshold was determined within the training dataset using the Youden Index derived from ROC analysis. The selected threshold was subsequently applied to the internal holdout test set to generate binary predictions and calculate classification metrics. Empirically derived classification thresholds for the individual biomarkers (procalcitonin, CRP, NLR, and P-lactate) and SIRS were likewise determined exclusively in the training dataset using the Youden Index and were then applied unchanged to the internal holdout test set for calculation of accuracy, sensitivity, specificity, and *F*1-score.

For clinical benchmarking, the developed machine learning models were additionally compared with conventional sepsis assessment approaches, including SIRS and individual biomarkers routinely used in sepsis evaluation (procalcitonin, CRP, NLR, and P-lactate). SIRS was selected as the primary clinical score comparator because all variables required for its calculation were available in the source dataset, whereas other commonly used clinical scores could not be fully reconstructed because required variables were unavailable. To evaluate the incremental predictive value of combining biomarkers, the primary biomarker-only logistic regression model included procalcitonin, P-lactate, and NLR, representing complementary inflammatory and metabolic markers commonly evaluated in sepsis assessment. All three biomarkers were entered simultaneously into the multivariable logistic regression model without data-driven variable selection. Multicollinearity among these biomarkers was assessed in the training dataset using variance inflation factors (VIFs). Because CRP was also routinely available and clinically relevant, an expanded biomarker-only model additionally including CRP was evaluated as a sensitivity analysis.

To assess the robustness of the feature-selection strategy, all four machine learning models were additionally refitted using all 13 prespecified admission-stage candidate predictors without LASSO-based feature selection. These sensitivity models retained the same train–test split, five-fold cross-validation structure, hyperparameter search spaces, and AUROC-based model-selection criterion as the primary analysis. Results of these sensitivity analyses are presented in Supplementary Table S1.

Model calibration was assessed using calibration plots, the Brier score, calibration intercept, and calibration slope. Calibration plots were generated by comparing observed and predicted event probabilities across risk groups. Calibration intercept and slope were estimated in the internal holdout test set for models generating predicted probabilities, with ideal values of 0 and 1, respectively.

The clinical utility of the developed models was evaluated using DCA. Net benefit was calculated across threshold probabilities from 0.01 to 0.99 for all machine learning models and the biomarker-only logistic regression model, with comparison against treat-all and treat-none strategies. Interpretation focused on the 0.20–0.40 range, which was selected as a clinically plausible low-to-moderate risk range for decision support in patients already presenting with suspected sepsis, reflecting a setting in which the consequences of missed sepsis may warrant additional clinical assessment at predicted probabilities below 50%.

To quantify statistical uncertainty, bootstrap resampling with 1000 iterations was performed on the internal holdout test set. Bootstrap-derived 95% confidence intervals (CIs) were calculated for all reported performance metrics.

Comparisons between AUROC values of competing models were performed using DeLong's test for paired ROC curves. Pairwise comparisons were conducted among the developed machine learning models and between machine learning models, conventional biomarkers, SIRS, and the biomarker-only logistic regression model.

### Explainability analysis

Model interpretability was evaluated using complementary model-intrinsic and *post hoc* explanation approaches. First, an EBM was developed as an inherently interpretable model within the same modeling framework. EBM, a generalized additive model-based machine learning approach, enables nonlinear predictor effects to be estimated while preserving feature-level transparency. Global feature importance rankings and feature effect plots derived from the EBM were used to identify influential predictors and visualize their individual contributions to the predicted probability of sepsis.

Second, *post hoc* explainability was performed for the LightGBM model, which achieved the numerically highest AUROC and accuracy in the internal holdout test set. SHAP values were used to quantify predictor contributions to LightGBM predictions. Global SHAP importance was calculated using mean absolute SHAP values. SHAP summary (beeswarm) plots were generated to visualize the magnitude and direction of predictor effects across individual observations, whereas SHAP dependence plots were used to investigate potential nonlinear relationships between predictor values and model predictions. Rug marks representing the distribution of observed predictor values in the internal holdout test set were added to the SHAP dependence plots to facilitate assessment of data density across the predictor range. To provide explanations that reflected model behavior on previously unseen data, all SHAP analyses were performed using predictions generated from the internal holdout test set.

Together, EBM and SHAP provided complementary interpretability perspectives, with EBM offering transparent model-intrinsic explanations and SHAP enabling *post hoc* interpretation of the LightGBM model.

### Statistical analysis

The distribution of continuous variables was assessed using the Shapiro–Wilk test. Normally distributed variables were summarized as mean ± standard deviation, whereas non-normally distributed variables were presented as median (25th–75th percentiles). Categorical variables were reported as frequencies and percentages.

Comparisons between the sepsis and non-sepsis groups were performed using the independent samples *t*-test or Mann–Whitney *U* test for continuous variables and the chi-square test for categorical variables, as appropriate. AUROCs of the primary and corresponding sensitivity models were compared using paired DeLong tests. For the primary pairwise AUROC comparisons, Holm correction was applied to account for multiple testing across comparisons among the developed machine learning models and between each machine learning model and the reference comparators. Holm correction was also applied to the four paired comparisons between the 11- and 13-predictor machine learning models.

All analyses were performed in R version 4.5.2 (R Foundation for Statistical Computing, Vienna, Austria) using RStudio (Posit Software, PBC, Boston, MA, USA). Model development and evaluation were conducted using the R packages *caret*, *glmnet*, *pROC*, *PRROC*, *xgboost*, and *dcurves*. The Explainable Boosting Machine (EBM), LightGBM, and SHAP analyses were implemented through the *reticulate* interface using the Python packages *interpret*, *lightgbm*, and *shap*, respectively. A fixed random seed (123) was used throughout model development, validation, and bootstrap resampling to ensure reproducibility. Statistical significance was defined as a two-sided *p*-value < 0.05. No formal sample size calculation was performed because this was a secondary analysis of a fixed publicly available cohort.

## Results

### Study population and baseline characteristics

A total of 1,572 patients were included in the study. After stratified random partitioning, 1,101 patients were assigned to the training set and 471 patients to the internal holdout test set. The prevalence of sepsis was maintained across datasets, accounting for 35.6% of patients in the training set and 35.7% in the test set.

Baseline characteristics of patients in the training and test datasets are presented in Table 1. In both datasets, patients with sepsis were older than those without sepsis (both *p* < 0.001). Compared with the non-sepsis group, patients with sepsis generally exhibited higher respiratory rates, lower oxygen saturation levels, higher leukocyte particle concentration, and increased levels of inflammatory and metabolic biomarkers, including CRP, procalcitonin, NLR, and P-lactate. Similar patterns were observed in both datasets.

**Table 1:** Baseline characteristics of patients with and without sepsis in the training and test datasets.

	Training set (*N* = 1,101)			Test set (*N* = 471)		
	Non-sepsis (*n* = 709)	Sepsis (*n* = 392)	*p*	Non-sepsis (*n* = 303)	Sepsis (*n* = 168)	*p*
Age (years)	66 (51.50–78)	77 (66–85)	**<0** **.** **001**	70 (58–79)	76 (68.25–83)	**<0** **.** **001**
Sex (male)	387 (54.6)	205 (52.3)	0.466	182 (60.1)	101 (60.1)	0.991
SBP (mmHg)	136.50 (120–153)	132 (111–150)	**0** **.** **009**	136.43 ± 24.91	128.30 ± 24.60	**<0** **.** **001**
Respiratory rate (breaths/min)	22 (18–28)	24 (20–30)	**<0** **.** **001**	22 (19–26)	26 (24–32)	**<0** **.** **001**
Oxygen saturation (%)	96 (94–97)	94 (90–96)	**<0** **.** **001**	95 (94–97)	94 (90–96)	**<0** **.** **001**
Heart rate (beats/min)	96 (84–109)	99 (85–112.50)	**0** **.** **030**	93 (80–104)	99.50 (84–113)	**0** **.** **002**
Body temperature (°C)	37.90 (37–38.60)	38 (37.20–38.70)	0.205	37.70 (37–38.45)	38.10 (37.10–38.80)	**0** **.** **004**
Hemoglobin (g/L)	131 (119–143)	127 (116.75–140)	**0** **.** **022**	132 (120–145)	129 (111.75–140)	**0** **.** **014**
Leukocyte particle concentration (×10^9 ^cells/L)	11.20 (8.20–14.43)	13.20 (9.90–17.90)	**<0** **.** **001**	11.50 (8.10–15.20)	12.90 (9.60–16.78)	**0** **.** **005**
CRP (mg/L)	89 (38–157)	111.50 (47–193.25)	**<0** **.** **001**	96 (35–160.50)	145 (65–217)	**<0** **.** **001**
Procalcitonin (ng/mL)	0.14 (0.05–0.56)	0.46 (0.13–4.43)	**<0** **.** **001**	0.12 (0.05–0.57)	0.62 (0.13–3.65)	**<0** **.** **001**
NLR	8 (4.80–14.40)	13 (7.88–22.43)	**<0** **.** **001**	7.90 (4.40–14.08)	12.90 (8.50–22.40)	**<0** **.** **001**
P-lactate (mmol/L)	1.58 (1.20–2.10)	1.90 (1.47–2.72)	**<0** **.** **001**	1.61 (1.23–2.18)	1.91 (1.38–2.67)	**<0** **.** **001**

Data are presented as median (25th–75th percentiles), mean ± standard deviation, or *n* (%), as appropriate. Continuous variables were compared using the independent samples *t*-test or Mann–Whitney *U* test, whereas categorical variables were compared using the chi-square test. SBP, systolic blood pressure; CRP, C-reactive protein; NLR, neutrophil-to-lymphocyte ratio.

Bold values indicate statistical significance (*p* < 0.05).

### Missing data and imputation results

Missing values were present in several candidate predictors before imputation, but no variable showed excessive missingness. In the training set, the highest proportions of missing data were observed for respiratory rate (12.2%), body temperature (8.8%), oxygen saturation (6.0%), heart rate (5.8%), and SBP (5.4%). Similar patterns were observed in the internal holdout test set, where respiratory rate (10.6%), body temperature (10.2%), SBP (5.7%), oxygen saturation (5.5%), and heart rate (5.5%) had the highest missingness rates. Missingness in all remaining predictors was below 4%.

Twenty imputed datasets were generated using MICE. Imputation 14, identified as the representative completed dataset, was used for subsequent feature selection, model development, and internal holdout evaluation. Convergence diagnostics indicated adequate chain mixing, with no logged MICE events. Sensitivity analyses across five randomly selected completed datasets showed consistent model performance across imputations (Supplementary Table S2). AUROC values ranged from 0.751 to 0.756 for penalized logistic regression, 0.758–0.769 for XGBoost, 0.764–0.771 for LightGBM, and 0.760–0.769 for EBM, while the corresponding Brier-score ranges were 0.199–0.214, 0.184–0.187, 0.182–0.186, and 0.184–0.186, respectively. These findings indicate that the principal model-performance results were not materially dependent on the representative completed dataset used for the primary analysis.

### Feature selection results

Feature selection was performed using LASSO logistic regression within the training dataset. Five-fold cross-validation identified an optimal penalty parameter of *λ*_min_ = 0.00416. Of the 13 predefined candidate predictors, 11 retained non-zero coefficients and were selected for subsequent model development.

The retained predictors included age, sex, SBP, respiratory rate, oxygen saturation, body temperature, leukocyte particle concentration, CRP, procalcitonin, NLR, and P-lactate. Heart rate and hemoglobin were shrunk to zero and excluded from the final predictor set. The selected predictors were subsequently used for the development and validation of all machine learning models.

### Cross-validation and hyperparameter optimization

Hyperparameter optimization was performed using grid search combined with five-fold cross-validation within the training dataset. The optimal hyperparameter configurations and corresponding cross-validated discrimination performances are presented in Table 2.

**Table 2:** Optimal hyperparameter configurations and cross-validation performance of the machine learning models.

Model	Optimal hyperparameters	Mean AUROC ± SD
LR	*α* = 0, *λ* = 6.723	0.757 ± 0.017
EBM	max_bins = 64, learning_rate = 0.01, max_rounds = 500, interactions = 0	0.763 ± 0.024
XGBoost	eta = 0.05, max_depth = 2, subsample = 0.8, colsample_bytree = 0.8, min_child_weight = 1, nrounds = 100	0.765 ± 0.026
LightGBM	num_leaves = 7, learning_rate = 0.01, nrounds = 300, max_depth = 3, min_data_in_leaf = 20, feature_fraction = 1.0, bagging_fraction = 0.8	0.761 ± 0.026

LR, penalized logistic regression; EBM, Explainable Boosting Machine; XGBoost, Extreme Gradient Boosting; LightGBM, Light Gradient Boosting Machine; AUROC, area under the receiver operating characteristic curve; SD, standard deviation.

Mean cross-validated AUROC values ranged from 0.757 to 0.765 across the evaluated models (Table 2). Overall, all models demonstrated comparable discrimination performance during cross-validation and were therefore retained for final evaluation in the internal holdout test set.

### Performance on the internal holdout test set

The discrimination and classification performance of the machine learning models, conventional biomarkers, SIRS, and the biomarker-only logistic regression model in the internal holdout test set are summarized in Table 3, and corresponding ROC curves are presented in Figure 2.

**Table 3:** Discrimination, classification, and calibration performance of machine learning models, conventional biomarkers, and SIRS in the internal holdout test set.

Model	AUROC	Accuracy	Sensitivity	Specificity	*F*1-score	AUPRC	Brier score
SIRS	0.644 (0.596–0.691)	0.618 (0.575–0.660)	0.577 (0.503–0.647)	0.640 (0.587–0.696)	0.519 (0.454–0.577)	0.467 (0.402–0.539)	NA
CRP	0.625 (0.569–0.677)	0.616 (0.573–0.660)	0.560 (0.478–0.639)	0.647 (0.591–0.699)	0.509 (0.446–0.566)	0.478 (0.404–0.550)	NA
Procalcitonin	0.693 (0.644–0.742)	0.631 (0.588–0.675)	0.613 (0.539–0.682)	0.640 (0.586–0.691)	0.542 (0.480–0.599)	0.563 (0.482–0.641)	NA
NLR	0.681 (0.630–0.733)	0.643 (0.601–0.688)	0.595 (0.519–0.671)	0.670 (0.617–0.721)	0.543 (0.480–0.598)	0.488 (0.416–0.571)	NA
P-lactate	0.599 (0.543–0.654)	0.543 (0.499–0.586)	0.637 (0.564–0.709)	0.492 (0.438–0.550)	0.499 (0.438–0.555)	0.466 (0.393–0.548)	NA
Biomarker-only LR	0.710 (0.661–0.760)	0.639 (0.597–0.684)	0.679 (0.606–0.755)	0.617 (0.562–0.669)	0.573 (0.509–0.629)	0.543 (0.464–0.627)	0.210 (0.193–0.226)
LR	0.753 (0.705–0.801)	0.662 (0.618–0.709)	0.786 (0.722–0.851)	0.594 (0.534–0.650)	0.624 (0.568–0.676)	0.581 (0.520–0.652)	0.225 (0.212–0.236)
XGBoost	0.763 (0.715–0.813)	0.673 (0.628–0.715)	0.774 (0.708–0.839)	0.617 (0.558–0.673)	0.628 (0.574–0.681)	0.629 (0.567–0.692)	0.185 (0.168–0.202)
LightGBM	0.767 (0.721–0.815)	0.686 (0.643–0.728)	0.804 (0.744–0.863)	0.621 (0.568–0.680)	0.646 (0.595–0.699)	0.633 (0.573–0.697)	0.185 (0.168–0.200)
EBM	0.766 (0.720–0.814)	0.662 (0.620–0.705)	0.845 (0.786–0.899)	0.561 (0.505–0.617)	0.641 (0.595–0.690)	0.637 (0.577–0.698)	0.184 (0.168–0.200)

Values are presented as point estimates with bootstrap-derived 95% CIs. AUROC, area under the receiver operating characteristic curve; AUPRC, area under the precision-recall curve; LR, penalized logistic regression; EBM, Explainable Boosting Machine; XGBoost, Extreme Gradient Boosting; LightGBM, Light Gradient Boosting Machine; SIRS, Systemic Inflammatory Response Syndrome; NLR, neutrophil-to-lymphocyte ratio; CRP, C-reactive protein. Biomarker-only LR denotes a logistic regression model including only procalcitonin, P-lactate, and NLR. Brier score was not calculated for SIRS, Procalcitonin, P-lactate, NLR, or CRP because these approaches do not generate calibrated probability estimates. The biomarker-only LR model was included as a clinically relevant baseline comparator to evaluate the incremental predictive value of demographic and physiological variables beyond laboratory biomarkers alone.

**Figure 2:**
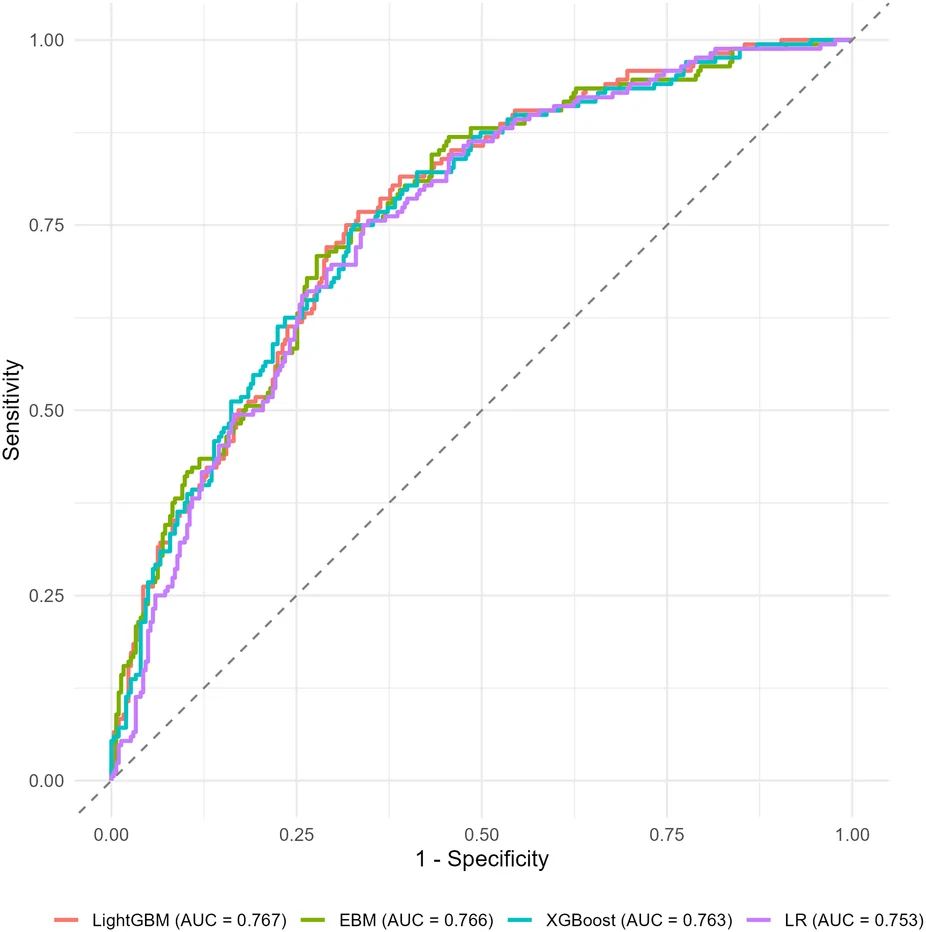
ROC curves of the developed machine learning models for identifying Sepsis-3 in the internal holdout test set. Receiver operating characteristic (ROC) curves of the developed machine learning models (LightGBM, XGBoost, EBM, and penalized logistic regression) for identifying Sepsis-3-defined sepsis in the internal holdout test set. The diagonal dashed line represents the performance of a non-informative classifier (AUROC = 0.50). LightGBM, Light Gradient Boosting Machine; XGBoost, Extreme Gradient Boosting; EBM, Explainable Boosting Machine; LR, penalized logistic regression; AUROC, area under the receiver operating characteristic curve.

Among the conventional comparators, procalcitonin demonstrated the highest discriminative ability (AUROC 0.693, 95% CI 0.644–0.742), followed by NLR (AUROC 0.681, 95% CI 0.630–0.733), SIRS (AUROC 0.644, 95% CI 0.596–0.691), CRP (AUROC 0.625, 95% CI 0.569–0.677), and P-lactate (AUROC 0.599, 95% CI 0.543–0.654). No evidence of problematic multicollinearity was observed among procalcitonin, NLR, and P-lactate, with VIFs of 1.052, 1.052, and 1.047, respectively. The biomarker-only logistic regression model, incorporating procalcitonin, P-lactate, and NLR, achieved an AUROC of 0.710 (95% CI 0.661–0.760), exceeding the performance of all individual biomarkers and SIRS.

All developed machine learning models demonstrated higher discrimination than the conventional comparators and the biomarker-only logistic regression model. AUROC values ranged from 0.753 (95% CI 0.705–0.801) for penalized logistic regression to 0.767 (95% CI 0.721–0.815) for LightGBM. LightGBM achieved the highest AUROC (0.767), accuracy (0.686), and F1-score (0.646), whereas EBM achieved the highest sensitivity (0.845, 95% CI 0.786–0.899) and AUPRC (0.637). Penalized logistic regression demonstrated the highest specificity (0.594) among the developed models.

Pairwise comparisons of AUROC values using DeLong's test revealed no statistically significant differences among the four developed machine learning models. After Holm correction for multiple comparisons, all four machine learning models retained significantly higher AUROC values than SIRS, CRP, and P-lactate (all Holm-adjusted *p* < 0.05). Among comparisons with the biomarker-only logistic regression model, only EBM remained statistically significant after multiplicity adjustment (Holm-adjusted *p* = 0.043). Differences between the machine learning models and procalcitonin or NLR were not statistically significant after Holm correction.

### Sensitivity analyses

Sensitivity analyses using all 13 prespecified admission-stage candidate predictors yielded discrimination comparable to that of the primary 11-predictor models. Test-set AUROC values were 0.757 for penalized LR, 0.763 for XGBoost, 0.769 for LightGBM, and 0.768 for EBM. Paired DeLong comparisons between each primary 11-predictor model and its corresponding 13-predictor sensitivity model showed no statistically significant differences in AUROC (all Holm-adjusted *p* > 0.05). The expanded biomarker-only logistic regression model incorporating procalcitonin, NLR, P-lactate, and CRP achieved an AUROC of 0.723 (95% CI 0.673–0.769), compared with 0.710 (95% CI 0.661–0.760) for the primary three-biomarker model; this difference was not statistically significant (paired DeLong test, *p* = 0.169). Overall, inclusion of the two predictors excluded by LASSO and addition of CRP to the biomarker-only comparator did not materially alter the main findings (Supplementary Table S1).

### Calibration and clinical utility

Calibration plots for the evaluated models are presented in Figure 3. Calibration curves demonstrated good agreement between predicted and observed event probabilities for the biomarker-only logistic regression model, LightGBM, XGBoost, and EBM across most of the observed risk range. Calibration intercept and slope values were as follows: biomarker-only logistic regression, 0.037 and 1.040; XGBoost, −0.079 and 1.078; LightGBM, −0.079 and 1.160; EBM, −0.070 and 1.199; and penalized logistic regression, −0.002 and 18.217, respectively. Penalized logistic regression showed a markedly elevated calibration slope (18.217), together with a narrow range of predicted probabilities and visible deviation from the ideal calibration line. Despite its reasonable discrimination, penalized logistic regression was substantially miscalibrated, as reflected by the markedly elevated calibration slope and narrow range of predicted probabilities. Consistent with these findings, EBM achieved the lowest Brier score (0.184), followed closely by LightGBM (0.185) and XGBoost (0.185). The biomarker-only logistic regression model showed a higher Brier score (0.210), whereas penalized logistic regression demonstrated the highest Brier score (0.225), indicating lower overall probabilistic accuracy.

**Figure 3:**
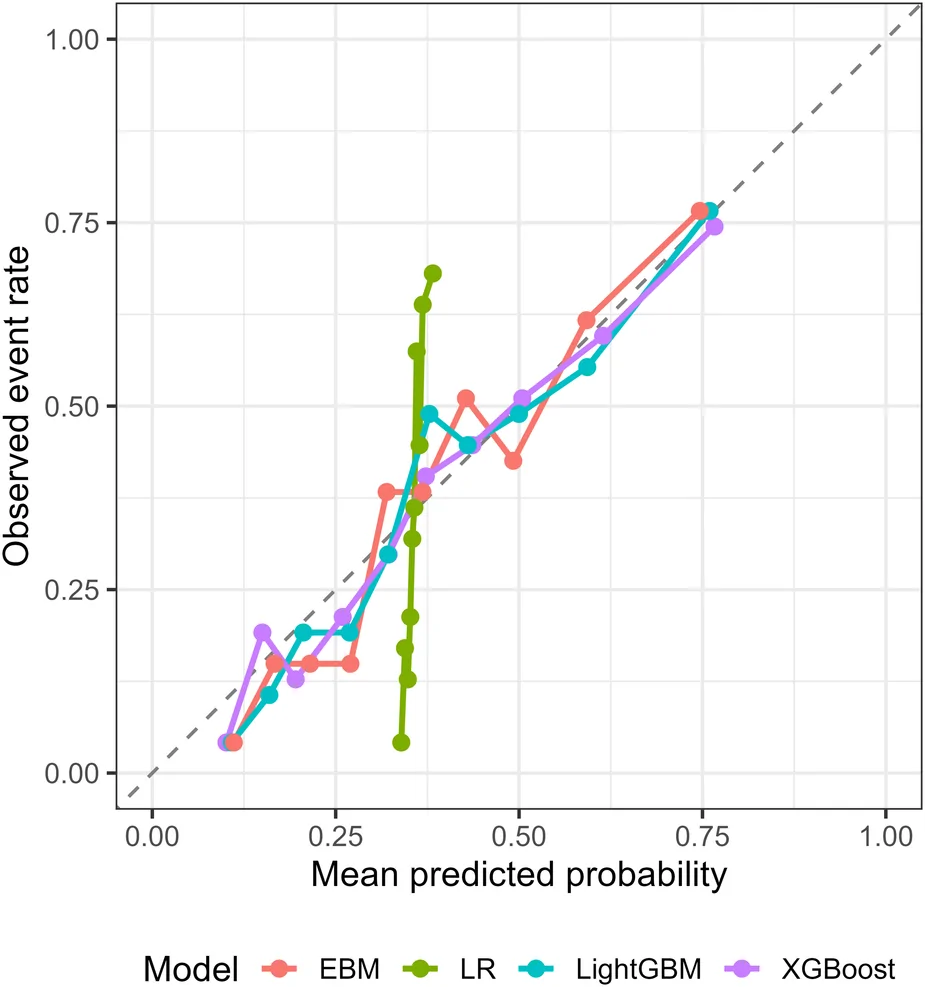
Calibration plots of machine learning models and the biomarker-only logistic regression model in the internal holdout test set. Calibration plots comparing mean predicted probabilities with observed event rates across risk groups in the internal holdout test set. The dashed diagonal line represents perfect calibration, indicating complete agreement between predicted and observed event probabilities. Curves closer to the diagonal line indicate better calibration performance. Calibration was evaluated for the biomarker-only logistic regression model and the developed machine learning models (penalized LR, XGBoost, LightGBM, and EBM). EBM, Explainable Boosting Machine; XGBoost, Extreme Gradient Boosting; LightGBM, Light Gradient Boosting Machine; LR, penalized logistic regression.

Decision curve analysis demonstrated positive net benefit for all developed machine learning models across clinically relevant threshold probabilities (Figure 4). LightGBM, XGBoost, and EBM exhibited largely overlapping decision curves and consistently provided greater net benefit than both penalized logistic regression and the biomarker-only logistic regression model across a broad range of threshold probabilities. Although the biomarker-only logistic regression model remained superior to the treat-all and treat-none strategies over part of the evaluated threshold range, its clinical utility was generally lower than that of the machine learning models. At higher threshold probabilities, the net benefits of all models gradually approached zero, indicating reduced clinical usefulness under increasingly restrictive decision thresholds.

**Figure 4:**
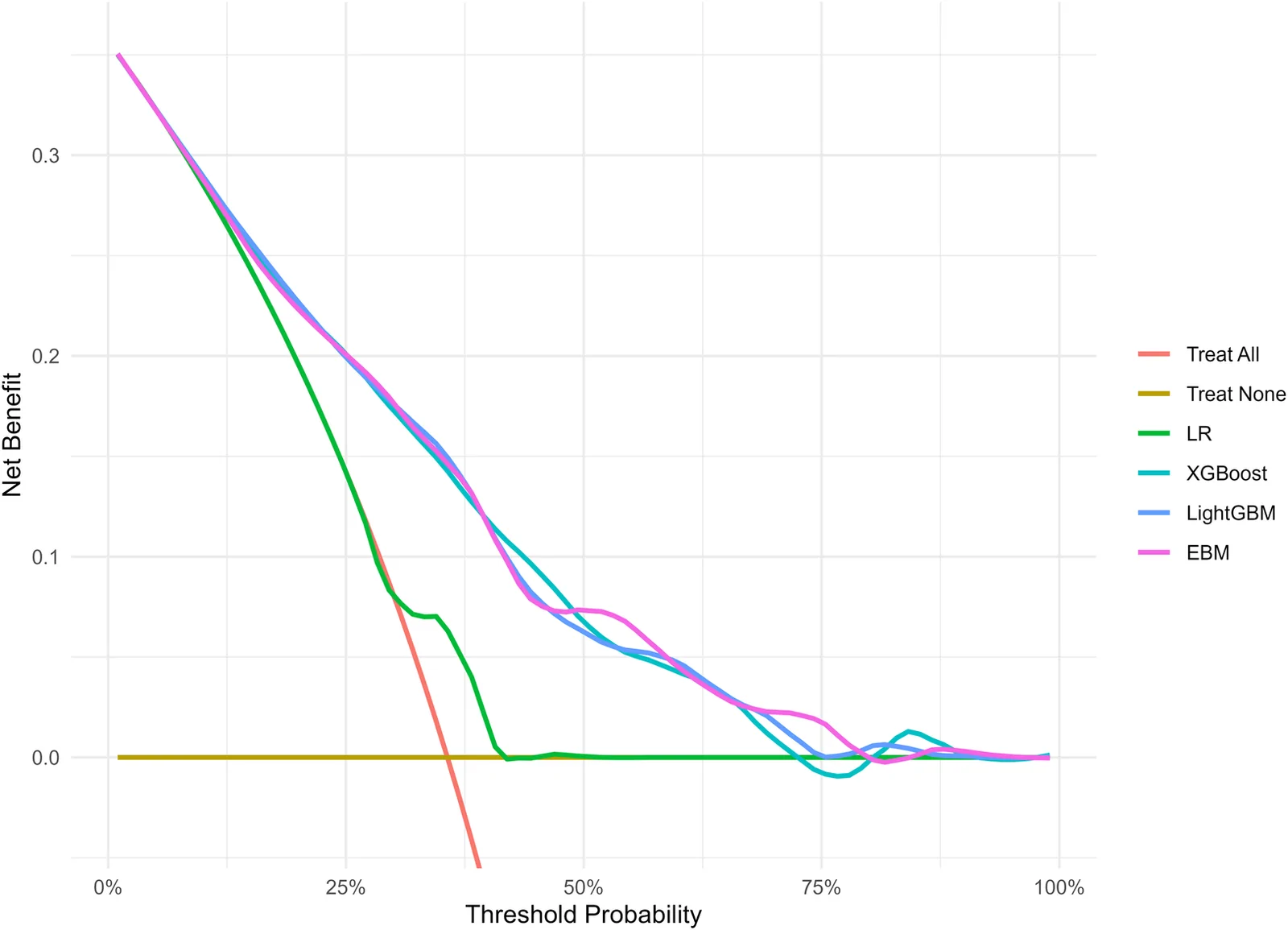
Decision curve analysis of machine learning models and the biomarker-only logistic regression model for Sepsis-3 identification in the internal holdout test set. Decision curve analysis comparing the decision-analytic net benefit of the developed machine learning models (LightGBM, XGBoost, EBM, and penalized logistic regression) and the biomarker-only logistic regression model across a range of threshold probabilities in the internal holdout test set. The biomarker-only logistic regression model included procalcitonin, P-lactate, and NLR. The “Treat All” and “Treat None” curves represent default clinical strategies of assuming all patients or no patients have sepsis, respectively. Higher net benefit indicates greater decision-analytic utility under the assumptions of DCA. LightGBM, Light Gradient Boosting Machine; XGBoost, Extreme Gradient Boosting; EBM, Explainable Boosting Machine; LR, penalized logistic regression.

### Explainability analysis

To improve model transparency and facilitate clinical interpretation, explainability analyses were performed using two complementary approaches. EBM was used as an intrinsically interpretable model to characterize global predictor effects, whereas SHAP values were calculated for LightGBM, which achieved the numerically highest AUROC and accuracy, to provide *post hoc* explanations of individual predictions. Global feature importance rankings derived from the EBM are presented in Figure 5. Age, procalcitonin, oxygen saturation, respiratory rate, and NLR emerged as the five most influential predictors of sepsis. Leukocyte particle concentration, SBP, P-lactate, and CRP demonstrated moderate importance, whereas body temperature and sex contributed relatively little to model predictions.

**Figure 5:**
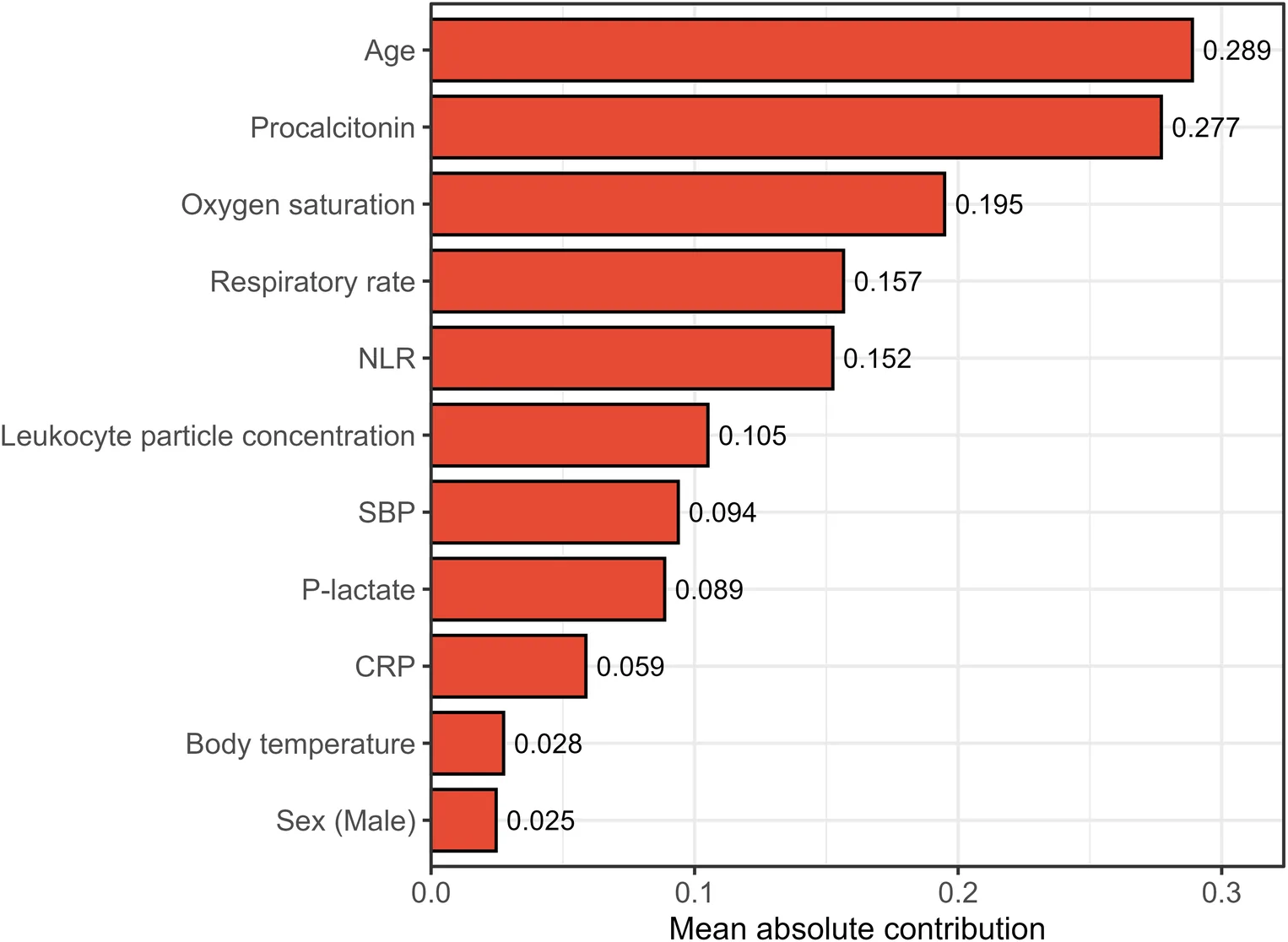
Global feature importance of predictors in the EBM model. Global feature importance rankings derived from the Explainable Boosting Machine (EBM). Importance values represent the mean absolute contribution of each predictor to the model predictions. Higher values indicate greater influence on predicted sepsis risk. NLR, neutrophil-to-lymphocyte ratio; SBP, systolic blood pressure; P-lactate, plasma lactate concentration; CRP, C-reactive protein.

To further characterize the relationships between predictor values and model predictions, feature effect plots were examined for the five most influential variables (Figure 6). Increasing age was associated with a progressively higher contribution to predicted sepsis risk, with a steeper model-estimated increase observed at older ages. Similarly, increasing procalcitonin concentrations were associated with a marked increase in predicted risk, although the effect gradually plateaued at higher values. Respiratory rate and NLR demonstrated positive associations with sepsis prediction, indicating increasing model contributions as their values increased. In contrast, oxygen saturation exhibited an inverse relationship, with lower saturation values contributing to higher predicted sepsis risk.

**Figure 6:**
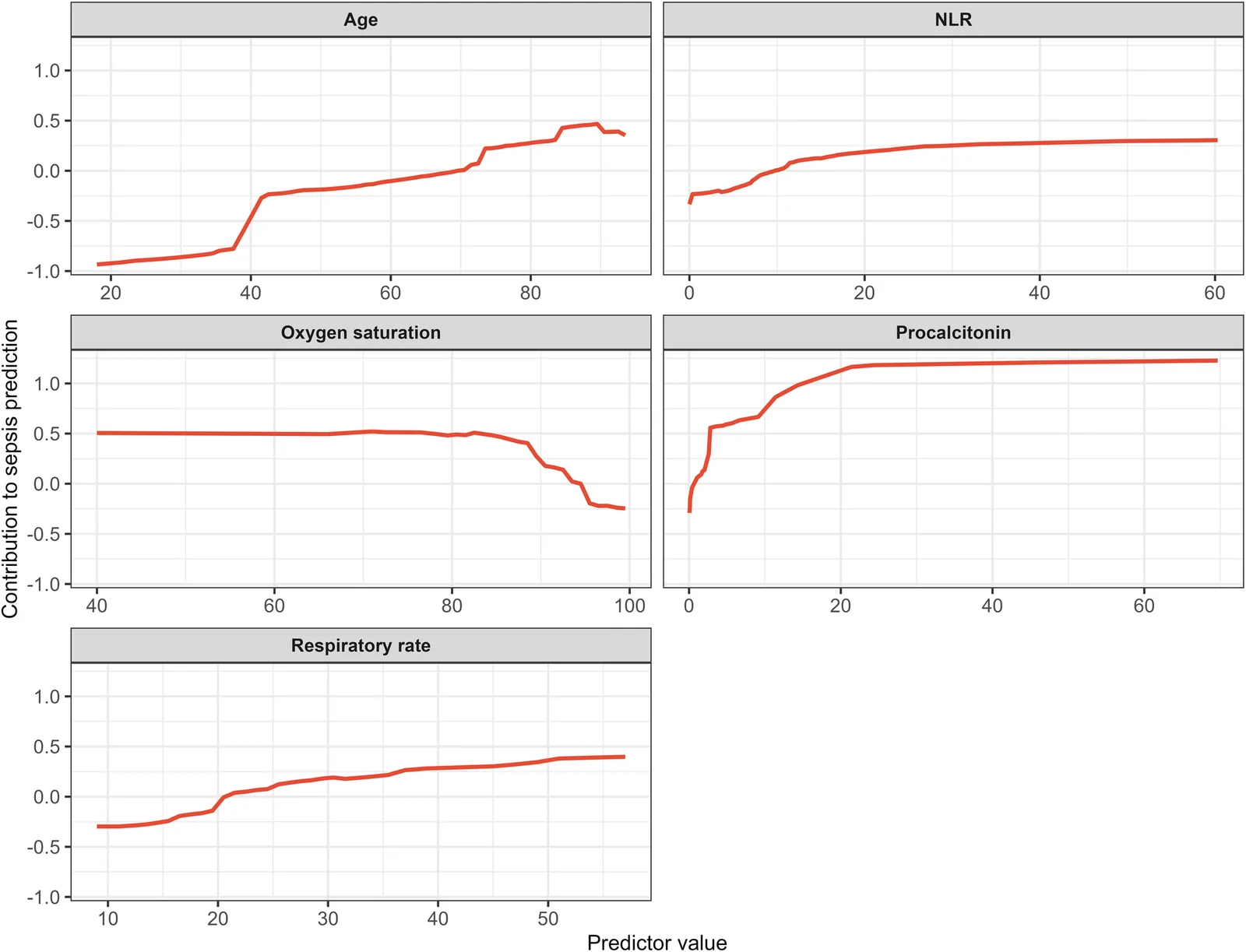
Feature effect plots for the five most influential predictors identified by the EBM. Feature effect plots illustrating the relationship between predictor values and their contribution to the EBM prediction score. Positive values indicate an increased contribution to predicted sepsis risk, whereas negative values indicate a decreased contribution. The plots are shown for the five most influential predictors identified by the EBM model: age, procalcitonin, oxygen saturation, respiratory rate, and neutrophil-to-lymphocyte ratio (NLR). The *x*-axis represents predictor values and the *y*-axis represents the contribution of each predictor to the EBM prediction score.

Overall, the EBM analyses demonstrated clinically plausible and nonlinear relationships between predictor values and model predictions. To determine whether similar predictor importance patterns were observed in LightGBM, SHAP analyses were subsequently performed for this model. Global SHAP importance rankings showed that age, procalcitonin, oxygen saturation, NLR, and respiratory rate were the most influential predictors, closely mirroring the feature importance rankings obtained from the EBM model (Figure 7).

**Figure 7:**
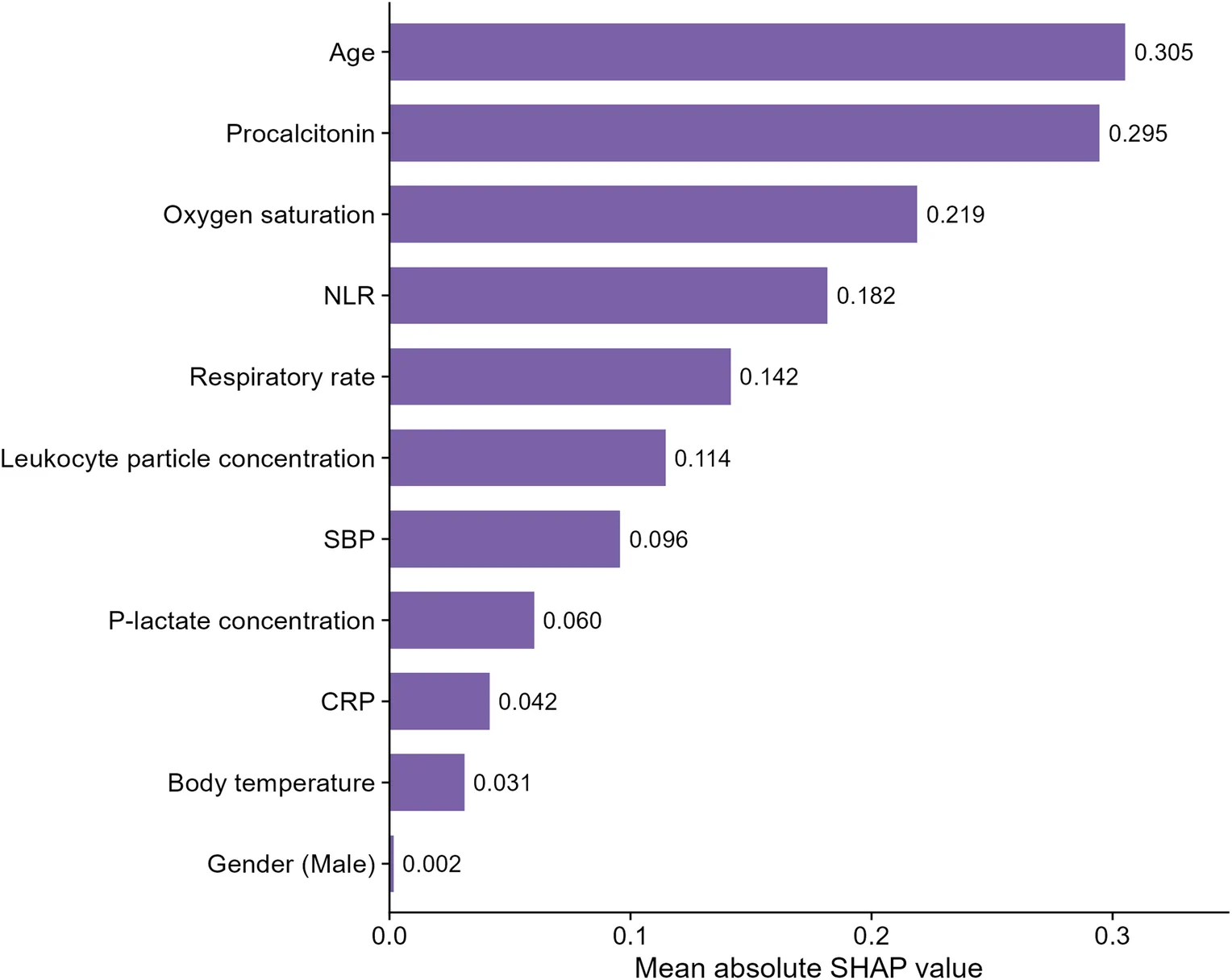
Global SHAP feature importance rankings for the LightGBM model. Global SHAP feature importance rankings derived from the Light Gradient Boosting Machine (LightGBM). SHAP values were calculated using the internal holdout test set. Importance values represent the mean absolute SHAP value for each predictor across all test observations. Higher values indicate greater overall influence on model predictions. Age, procalcitonin, oxygen saturation, NLR, and respiratory rate emerged as the most influential predictors. SHAP, SHapley Additive exPlanations; NLR, neutrophil-to-lymphocyte ratio; SBP, systolic blood pressure; CRP, C-reactive protein.

The SHAP summary plot (Figure 8) provided additional information regarding the direction of predictor effects. Higher values of age, procalcitonin, NLR, respiratory rate, leukocyte particle concentration, and P-lactate were generally associated with increased predicted sepsis risk. In contrast, lower oxygen saturation and lower SBP values were associated with higher SHAP values, indicating greater contributions to sepsis prediction.

**Figure 8:**
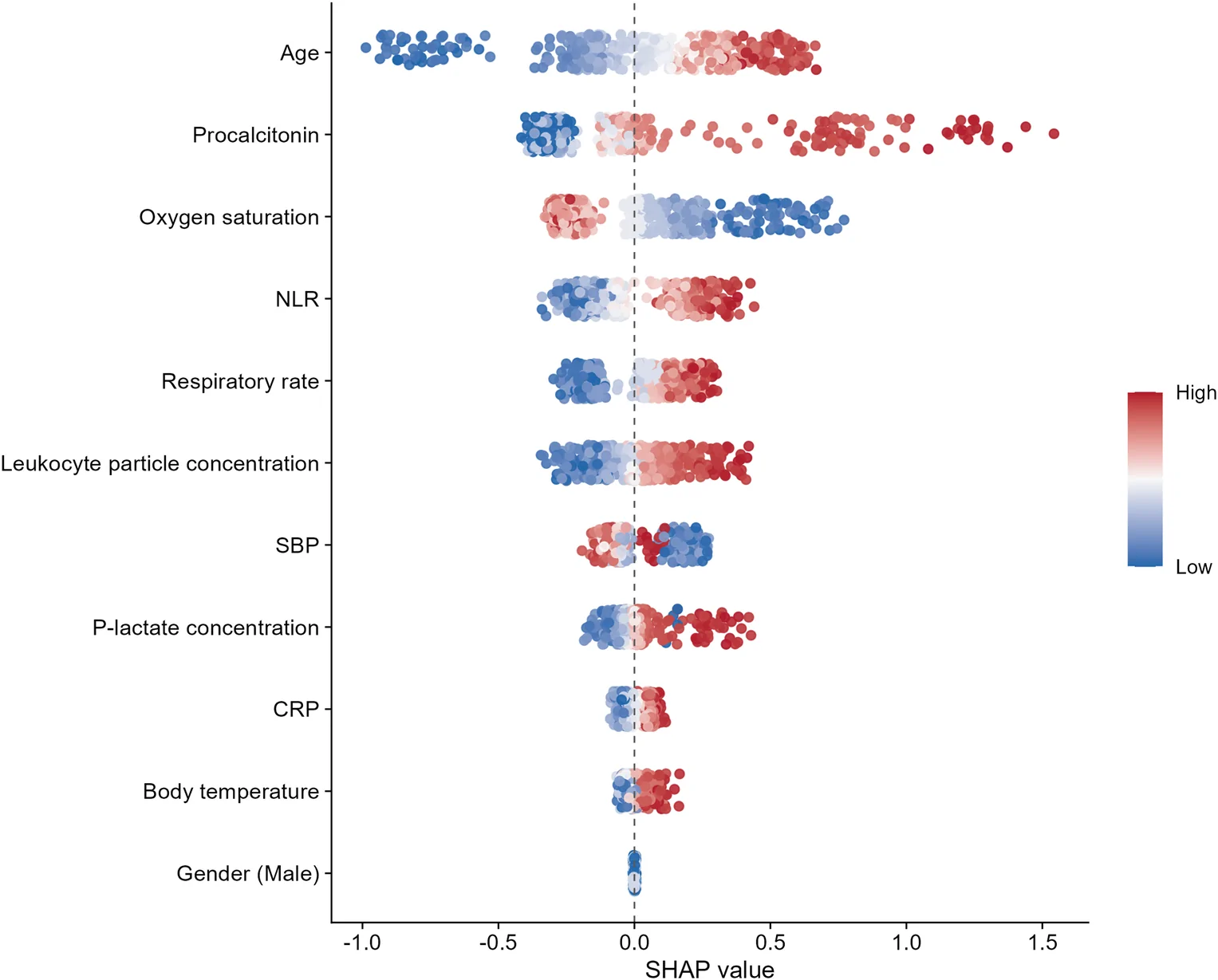
Shap summary plot for the LightGBM model. SHAP summary plot illustrating both the magnitude and direction of predictor effects in the LightGBM model using the internal holdout test set. Each point represents an individual patient from the test set. The *x*-axis shows SHAP values, indicating the contribution of a predictor to the model output. Positive SHAP values increase the predicted probability of sepsis, whereas negative SHAP values decrease it. Point color represents the original predictor value, with red indicating higher values and blue indicating lower values.

The SHAP dependence plots (Figure 9) revealed nonlinear predictor–response relationships for the most influential predictors. Model contributions generally increased at older ages, rose with increasing procalcitonin concentrations before tending to plateau at higher values, and increased with higher NLR and respiratory rate values. Conversely, lower oxygen saturation values were generally associated with greater contributions toward Sepsis-3 classification. These patterns represent model-estimated relationships and should not be interpreted as validated clinical thresholds.

**Figure 9:**
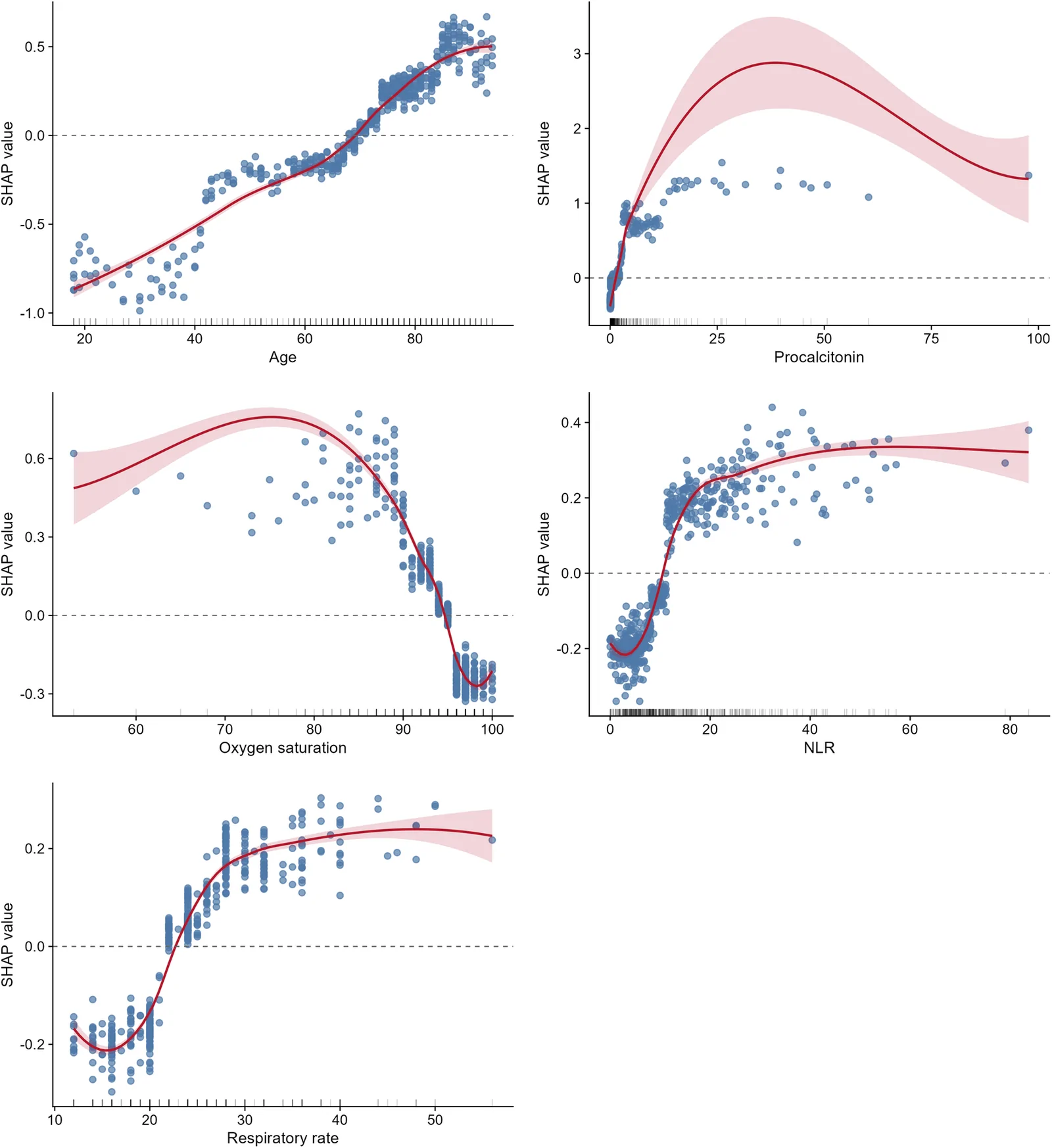
SHAP dependence plots for the five most influential predictors in the LightGBM model. SHAP dependence plots demonstrating the relationships between predictor values and their contributions to Sepsis-3 classification in the LightGBM model using the internal holdout test set. Each point represents an individual patient from the test set. The red curve indicates the smoothed trend of the predictor effect, and the shaded area represents the 95% CI. Positive SHAP values indicate increased predicted sepsis risk, whereas negative values indicate decreased risk. Rug marks along the *x*-axis indicate the distribution of observed predictor values in the internal holdout test set, allowing visual assessment of data density across the predictor range. The plots are shown for age, procalcitonin, oxygen saturation, NLR, and respiratory rate, which were identified as the most influential predictors by SHAP analysis.

## Discussion

In this secondary analysis of a prospective ED cohort, machine learning models based on routinely available admission-stage variables achieved AUROC values ranging from 0.753 to 0.767 for identifying Sepsis-3-defined sepsis. Discrimination did not differ significantly among the four developed machine learning models. LightGBM achieved the numerically highest AUROC and accuracy, whereas EBM provided the highest sensitivity and AUPRC while maintaining inherent interpretability. After Holm correction for multiple comparisons, all four machine learning models retained significantly higher discrimination than SIRS, CRP, and P-lactate, while only EBM remained significantly superior to the biomarker-only logistic regression model. Sensitivity analyses using all 13 prespecified candidate predictors yielded nearly unchanged discrimination across all four algorithms, indicating that the overall findings were robust to the LASSO-based feature-selection step (Supplementary Table S1). In addition to improved discrimination, XGBoost, LightGBM, and EBM demonstrated lower Brier scores and higher decision-analytic net benefit than the biomarker-only approach within the selected threshold range, suggesting that the integration of demographic, physiological, and laboratory information available at ED admission may provide additional predictive information beyond conventional biomarkers alone. These findings suggest that interpretable AI models based exclusively on routinely available admission-stage variables may support early sepsis risk stratification among ED patients presenting with suspected community-onset sepsis. The modest specificity observed for the highest-performing models indicates that their use as stand-alone diagnostic tools could result in a substantial number of false-positive classifications. In practice, these models would be more appropriately implemented as clinical decision-support tools rather than as autonomous diagnostic systems. Classification thresholds could be adjusted according to the intended clinical use, with higher thresholds increasing specificity at the expense of sensitivity. Therefore, threshold selection should be prospectively calibrated to the clinical context and evaluated in implementation studies to balance missed cases against unnecessary alerts, investigations, or treatment.

From an implementation perspective, the use of routinely collected structured admission variables may facilitate future integration of the model into real-time EHR-based clinical decision-support workflows. Such integration could allow automated calculation of patient-level sepsis risk without requiring additional data collection. However, the present study did not evaluate real-time deployment or its effect on clinician behavior or patient outcomes. Before clinical implementation, the model should undergo prospective external validation, local calibration and workflow testing, followed by impact studies assessing effects on clinician decision-making, treatment timing, escalation of care, alert burden, and patient-centered outcomes.

The observed predictor patterns were broadly consistent with clinical expectations and were identified across both explainability frameworks. Despite their different methodological foundations, the inherently interpretable EBM and the *post hoc* SHAP analysis of the LightGBM model highlighted largely the same predictors, with age, procalcitonin, oxygen saturation, respiratory rate, and NLR emerging as the most influential variables. These findings are consistent with established clinical and pathophysiological features of Sepsis-3, including host vulnerability, dysregulated inflammation, respiratory compromise, hemodynamic instability, and metabolic stress (3, 4). Importantly, the concordance between EBM and SHAP indicates that similar predictor-importance patterns were identified across two complementary interpretability frameworks (15, 22). However, these analyses describe how the fitted models use the predictors and should not be interpreted as evidence of causal relationships, independent biological importance, or the absence of algorithm-specific artifacts. In addition, nonlinear patterns at the extremes of predictor distributions should be interpreted cautiously because relatively sparse observations in these regions may influence the smoothed SHAP dependence curves. The predictor-importance patterns identified by EBM and SHAP should be interpreted as model-specific findings derived from the present cohort and may not generalize unchanged across different patient populations, institutions, or geographic settings. External validation in contemporary, geographically diverse cohorts is therefore required, together with reassessment of calibration, decision thresholds, and predictor-importance patterns before clinical implementation. The findings of the present study should be interpreted in the context of the original Skaraborg Hospital biomarker investigation, which evaluated procalcitonin, NLR, CRP, and P-lactate individually and in biomarker combinations among ED patients with suspected sepsis (8). In that study, procalcitonin achieved the highest discrimination for Sepsis-3-defined bacterial sepsis (AUROC 0.68), while combinations of biomarkers provided little additional diagnostic value over the best-performing individual biomarkers (8). Consistent with these observations, procalcitonin and NLR also emerged as important predictors in our models, and the biomarker-only logistic regression model incorporating procalcitonin, NLR, and P-lactate outperformed each individual biomarker. In a sensitivity analysis, expanding the biomarker-only model to additionally include CRP modestly increased its AUROC from 0.710 to 0.723; however, its discrimination remained below that of all four multivariable models, and the overall conclusions were unchanged (Supplementary Table S1). Although all four developed machine learning models showed numerically higher AUROC values than the primary biomarker-only model, only EBM remained statistically significant after Holm correction. These numerical differences suggest that routinely available clinical information may provide additional predictive information beyond laboratory biomarkers alone. By integrating routinely available demographic characteristics, vital signs, and laboratory measurements obtained at ED admission, the proposed models captured complementary predictive information that was not available to biomarker-only approaches. Importantly, these gains were achieved using a clinically feasible variable set, with favorable calibration and decision-analytic net-benefit findings observed particularly for XGBoost, LightGBM, and EBM, supporting the value of combining biomarker data with routinely collected clinical information for early identification of Sepsis-3-defined sepsis in the ED.

Our findings are broadly consistent with those reported by Yagin et al., who recently developed explainable machine learning models for sepsis prediction using the same prospective ED cohort and identified positive blood culture, oxygen saturation, and procalcitonin as the most influential predictors in their Explainable Boosting Machine (EBM) model. They reported an AUROC of 0.848 after applying SMOTE-NC resampling and repeated model development procedures. Similar to their results, age, oxygen saturation, procalcitonin, respiratory rate, and NLR consistently emerged among the most influential predictors in our EBM and SHAP analyses, showing broadly similar predictor-importance patterns across the two analyses. Accordingly, the present study should be interpreted as a clinically focused methodological reanalysis of the Skaraborg cohort rather than as validation in a new patient population. However, several methodological differences should be considered when comparing the reported performances. Yagin et al. employed Random Forest imputation, SMOTE-NC oversampling, and included variables such as positive blood culture and intensive care unit status, whereas our study used multiple imputation by chained equations (MICE), and focused exclusively on variables available during the initial ED assessment. Importantly, positive blood culture results and intensive care unit admission are generally unavailable at the time of triage and therefore may not be suitable for real-time prediction. By restricting predictors to routinely available demographic, physiological, and laboratory variables, our models were designed to better reflect real-world ED implementation. Despite these more conservative modeling assumptions, the models achieved moderate discrimination while relying exclusively on information available during the initial ED assessment.

This conservative design is also relevant when compared with broader ED sepsis prediction studies. Machine learning-based sepsis risk scores and XGBoost models have been developed for ED sepsis identification and compared with conventional sepsis and early-warning scores, including SIRS, SOFA, qSOFA, MEWS, and NEWS (10, 11). More recently, an interpretable emergency triage model based on structured electronic medical records showed that incorporating vital signs together with demographic characteristics, medical history, and chief complaints improved sepsis prediction compared with vital signs alone, with Gradient Boosting achieving an AUROC of 0.83; SHAP and LIME were used to enhance model interpretability (23). These studies support the value of machine learning for ED sepsis stratification, but many models rely on broad EHR data, unstructured text, or local operational definitions. The contribution of the present analysis is therefore clinically focused, emphasizing a restricted set of routinely available admission-stage predictors, avoidance of outcome-proximal information, and comparison of multiple algorithms within a consistent analytical framework. Rather than maximizing predictive performance through extensive feature engineering or data augmentation strategies, the present study also evaluated classification performance, calibration, and clinical utility in addition to discrimination, thereby providing a more comprehensive assessment of potential clinical applicability.

Natural language processing–based approaches have demonstrated that unstructured triage documentation contains substantial predictive information beyond routinely collected structured variables. The incorporation of free-text chief complaints and nursing triage assessments has been shown to increase discrimination for infection detection from an AUROC of 0.67 using demographic and vital sign data alone to 0.86 when free-text information was included (12). More recently, a transformer-based sepsis prediction model combining nursing triage notes and structured clinical variables from more than one million emergency department encounters achieved an AUROC of 0.94 with sensitivity and specificity of 0.87 and 0.85, respectively (13). These findings suggest that free-text documentation captures clinically relevant contextual information that may not be fully represented by structured variables alone. However, implementation of such models generally requires mature EHR infrastructure, standardized clinical documentation, and locally developed NLP pipelines. In contrast, our study intentionally focused on routinely available structured demographic, physiological, and laboratory variables. Although this design may yield lower discrimination than large-scale NLP-enhanced systems, it offers a simpler, more transparent, and potentially more portable framework for further evaluation in ED populations with suspected sepsis. The study also complements ICU-based sepsis prediction literature. Interpretable and explainable machine learning models have predicted sepsis onset before clinical recognition using ICU data (24, 25). An interpretable real-time model based on three-hour dynamic physiological indicators similarly reported an AUROC above 0.76 and used LIME for local explanations, but it relied on continuously monitored physiological data rather than first-admission ED variables (26). However, ICU models are often developed using dense longitudinal monitoring data that differ from the sparse, time-pressured ED admission context. Our approach addresses this gap by focusing on information realistically available at first ED assessment. This distinction is clinically important because ED clinicians must frequently make treatment decisions before repeated measurements and evolving clinical trajectories become available. More broadly, recent reviews of sepsis prediction models have highlighted substantial heterogeneity in study populations, outcome definitions, predictor selection, validation strategies, reporting quality, and pathways to clinical implementation (27, 28).

Several methodological strengths deserve emphasis. Preprocessing, feature selection, hyperparameter tuning, threshold determination, and model training were confined to the training set, reducing the risk of information leakage. The original class distribution was preserved without synthetic oversampling or undersampling, thereby avoiding alteration of the observed outcome prevalence. Model evaluation was not limited to AUROC or accuracy; sensitivity, specificity, *F*1-score, AUPRC, Brier score, calibration plots, bootstrap CIs, DeLong comparisons, and DCA were also considered. In addition, model performance was evaluated not only against individual biomarkers and SIRS but also against a biomarker-only logistic regression model, enabling assessment of the incremental predictive value provided by demographic and physiological variables beyond laboratory biomarkers alone. This is important because prediction models should be judged not only by discrimination but also by calibration, clinical usefulness, and transparent reporting (18, 29). The positive net benefit of the machine learning models across low-to-moderate threshold probabilities supports their potential value as decision-support tools rather than stand-alone diagnostic instruments (18).

This study has limitations. It was a secondary analysis of a historical single-center Swedish cohort, and prospective, geographically diverse external validation is therefore required. Because the cohort was collected between 2011 and 2012, temporal changes in ED workflows, sepsis management, laboratory testing, and population characteristics may affect transportability to contemporary practice. The source cohort consisted exclusively of patients already presenting with suspected community-onset sepsis and therefore does not represent an undifferentiated ED population. Accordingly, the observed prevalence, calibration, predictive values, and decision-curve findings may differ substantially in an unselected ED population, and the present results should not be interpreted as evidence supporting general ED-wide sepsis screening. The internal holdout test set was derived from the same original cohort and therefore represents internal holdout validation rather than external validation. In addition, the number of sepsis events in the internal holdout test set may have limited the ability to detect small differences in discrimination between the developed machine learning models. In addition, although multiple imputed datasets were generated, downstream machine-learning analyses were conducted using a single representative completed dataset; therefore, between-imputation variability was not propagated through the full modeling pipeline. Although the sensitivity analysis demonstrated broadly stable AUROC and Brier scores across randomly selected completed datasets, between-imputation uncertainty was not fully propagated through the primary modeling analysis. Accordingly, the reported confidence intervals reflect sampling variability within the representative completed dataset and do not incorporate the full between-imputation uncertainty. Comparison with other commonly used clinical scores was also limited by the absence of variables required for their complete calculation, including mental-status information and supplemental oxygen use. Finally, the intentionally restricted predictor set improved feasibility but may have reduced discrimination compared with models using microbiology, serial measurements, treatment variables, or free-text EHR data. Because the models were intentionally restricted to admission-stage measurements, they do not capture subsequent changes in physiological or laboratory variables that may reflect clinical deterioration, treatment response, or the evolving trajectory of sepsis. Incorporating serial measurements and temporal trends may therefore improve dynamic risk assessment and should be evaluated in future longitudinal studies.

## Conclusions

Machine learning models based on routinely available ED admission variables provided moderate discrimination and clinically relevant sensitivity, while XGBoost, LightGBM, and EBM additionally demonstrated favorable calibration and decision-curve findings for identifying Sepsis-3. LightGBM achieved the numerically highest AUROC and accuracy, whereas EBM offered the highest sensitivity and AUPRC with inherent interpretability. Compared with prior studies, the present modeling framework is deliberately conservative: it avoids positive blood culture and other outcome-proximal variables, preserves the original class distribution, evaluates calibration and decision-curve utility, and provides complementary EBM and SHAP explanations. These design choices support the potential of the framework as an interpretable AI-based CDS approach for Sepsis-3 identification among ED patients presenting with suspected community-onset sepsis. Future studies should externally and prospectively validate the model across diverse healthcare settings and determine whether integration into CDS workflows improves time to clinician review, antibiotic decision-making, escalation of care, and patient-centered outcomes.

## Data Availability

Publicly available datasets were analyzed in this study. This data can be found here: https://zenodo.org/records/823967 (30).
