# Supplementary material for: Interpretable admission-stage machine learning for early Sepsis-3 identification in the emergency department
Source: Front Med (Lausanne). 2026 Sep 18;13:1929426. doi: 10.3389/fmed.2026.1929426 (PMC13630677; doi:10.3389/fmed.2026.1929426)
Supplement: Supplementary file 1 [file Table1.docx]

**Supplementary Table S1.** Sensitivity analyses of machine learning models using all 13 candidate predictors and the expanded biomarker-only logistic regression model in the hold-out test dataset

| **Model** | **AUROC** | **Accuracy** | **Sensitivity** | **Specificity** | **F1-score** | **AUPRC** | **Brier score** |
| --- | --- | --- | --- | --- | --- | --- | --- |
| Expanded biomarker-only LR | 0.723  (0.673-0.769) | 0.675  (0.635-0.716) | 0.554  (0.483-0.622) | 0.743  (0.694-0.791) | 0.549  (0.484-0.607) | 0.560  (0.476-0.642) | 0.205  (0.189-0.220) |
| LR | 0.757  (0.711-0.805) | 0.682  (0.639-0.726) | 0.750  (0.684-0.817) | 0.644  (0.586-0.700) | 0.627  (0.570-0.685) | 0.590  (0.507-0.672) | 0.217  (0.205-0.228) |
| XGBoost | 0.763  (0.719-0.814) | 0.641  (0.597-0.690) | 0.815  (0.752-0.875) | 0.545  (0.487-0.605) | 0.619  (0.562-0.672) | 0.635  (0.557-0.715) | 0.187  (0.171-0.201) |
| LightGBM | 0.769  (0.726-0.817) | 0.696  (0.656-0.741) | 0.583  (0.511-0.661) | 0.759  (0.711-0.810) | 0.578  (0.513-0.643) | 0.634  (0.550-0.712) | 0.184  (0.168-0.199) |
| EBM | 0.768  (0.726-0.815) | 0.652  (0.607-0.696) | 0.863  (0.810-0.911) | 0.535  (0.475-0.592) | 0.639  (0.579-0.689) | 0.641  (0.560-0.721) | 0.184  (0.167-0.199) |

Values are presented as point estimates with bootstrap-derived 95% confidence intervals. The 13-predictor models were developed using all prespecified admission-stage candidate variables without LASSO-based feature selection, while retaining the same train–test split, five-fold cross-validation structure, hyperparameter search spaces, and model-selection criterion as in the primary analysis. The expanded biomarker-only logistic regression model included procalcitonin, neutrophil-to-lymphocyte ratio, plasma lactate, and C-reactive protein. AUROC, area under the receiver operating characteristic curve; AUPRC, area under the precision–recall curve; LR, penalized logistic regression; XGBoost, Extreme Gradient Boosting; LightGBM, Light Gradient Boosting Machine; EBM, Explainable Boosting Machine; NLR, neutrophil-to-lymphocyte ratio; CRP, C-reactive protein.
