# Supplementary material for: Interpretable admission-stage machine learning for early Sepsis-3 identification in the emergency department
Source: Front Med (Lausanne). 2026 Sep 18;13:1929426. doi: 10.3389/fmed.2026.1929426 (PMC13630677; doi:10.3389/fmed.2026.1929426)
Supplement: Supplementary file 2 [file Table2.docx]

**Supplementary Table S2.** Sensitivity analysis of model performance across randomly selected imputed datasets

| Imputation | LR AUROC | LR Brier | XGBoost AUROC | XGBoost Brier | LightGBM AUROC | LightGBM Brier | EBM AUROC | EBM Brier |
| --- | --- | --- | --- | --- | --- | --- | --- | --- |
| Imputation 14 | 0.753 | 0.225 | 0.763 | 0.185 | 0.767 | 0.185 | 0.766 | 0.184 |
| Imputation 1 | 0.756 | 0.201 | 0.769 | 0.184 | 0.771 | 0.182 | 0.769 | 0.184 |
| Imputation 3 | 0.751 | 0.202 | 0.761 | 0.186 | 0.767 | 0.184 | 0.762 | 0.186 |
| Imputation 7 | 0.753 | 0.213 | 0.758 | 0.187 | 0.764 | 0.185 | 0.760 | 0.186 |
| Imputation 10 | 0.751 | 0.199 | 0.764 | 0.186 | 0.765 | 0.186 | 0.761 | 0.186 |
| Imputation 17 | 0.752 | 0.214 | 0.761 | 0.186 | 0.765 | 0.185 | 0.764 | 0.185 |

AUROC, area under the receiver operating characteristic curve; LR, Penalized logistic regression; EBM, Explainable Boosting Machine. Imputation 14 corresponds to the representative completed dataset used for the primary analysis. For the sensitivity analysis, five completed datasets (Imputations 1, 3, 7, 10, and 17) were randomly selected from the 20 imputed datasets and the full modeling pipeline was repeated in each. The sensitivity analysis was performed to assess the stability of model performance across alternative completed datasets.
